# A late systemic and brain metastasis from subcutaneous leiomyosarcoma of the right forearm: a case report and review of the literature

**DOI:** 10.1186/s13256-020-02625-0

**Published:** 2021-01-19

**Authors:** Eric Dietel, Ulf Nestler, Hans Nenning, Christian Eisenlöffel, Ruth Stassart, Jürgen Meixensberger

**Affiliations:** 1 Department of Neurosurgery, University of Leipzig, Liebigstraße 20, 04103 Leipzig, Germany; 2 Elsapark Pathology associates, Elsastraße 1, 04315 Leipzig, Germany; 3 Department of Neuropathology, University of Leipzig, Liebigstraße 26, 04103 Leipzig, Germany

**Keywords:** Leiomyosarcoma, Cerebral metastasis, Radiotherapy

## Abstract

**Background:**

Leiomyosarcomas are rare malignant tumors which originate from smooth muscle cells and very seldom give rise to intracerebral metastases. Nearly all cases of intracranial metastases stem from leiomyosarcomas of the uterus. We present a 61-year-old Caucasian man who developed multiple intracranial and extracranial metastases from leiomyosarcoma of the right forearm, diagnosed and treated 9 years before the current presentation.

**Case presentation:**

The Caucasian patient presented to the emergency department due to a progressive hemiparesis on the left side. Magnetic resonance imaging scans of the neurocranium showed multiple intracerebral masses with perifocal edema. One of these was located in the right parietal lobe, corresponding to the hemiparesis. The patient underwent microsurgical complete resection of the parietal mass and was subsequently subjected to further radiotherapy. Histopathological studies revealed metastasis of the former leiomyosarcoma.

**Conclusions:**

Leiomyosarcomas represent a rare entity of mesenchymal tumors. Intracerebral metastasis of these tumors is even less frequent. This case shows the importance of long-term follow-up in patients with leiomyosarcoma.

## Background

Leiomyosarcomas are rare mesenchymal tumors which originate from smooth muscle cells. These tumors occur in all age groups and account for 10% of all soft tissue sarcomas [1]. Most commonly, leiomyosarcomas originate from the uterus, retroperitoneum, or large blood vessels. Prognosis depends on complete surgical excision and adjuvant radiotherapy. Nevertheless, about 40% of all leiomyosarcomas develop a local recurrence or metastasis [1]. In these cases, further therapeutic options are limited, and mostly palliative chemotherapy is administered.

In this report, we present the case of delayed intra- and extracerebral metastases of a non-uterine leiomyosarcoma and review the literature concerning clinical management adopted and potential novel therapies.

## Case presentation

A 61-year-old Caucasian male patient presented with progressive hemiparesis of the left side, which started in the left leg and ascended to the left arm. A seizure was not reported. Magnetic resonance imaging (MRI) scans of the head showed multiple intracerebral mass lesions.

Leiomyosarcoma of the right forearm had been diagnosed 9 years earlier after partial resection (R2) of a growing tumor mass. Subsequently, the patient had declined a follow-up resection or further adjuvant treatment. Due to the tumor's progressive growth, the patient was readmitted 1 year after the first diagnosis via the surgical outpatient section. On presentation, the patient had normal weight and appeared well. A cardiac exam showed a regular rate and no murmur. A pulmonary exam showed a vesicular breath murmur. The examination of the right forearm showed a rigid tumor. The neurological exam revealed no abnormalities. Laboratory test results were normal (leukocytes: 8.3 ×10^9^/l, hemoglobin: 9.5 mmol/l, thrombocytes: 269 ×10^9^/l, creatinine: 80.0 µmol/l). The daily medication was pantoprazole 40 mg (0-0-1) and diclofenac 50 mg (1-1-1). Besides the leiomyosarcoma, the past medical history of the patient was empty. The patient did not smoke or consume alcohol in excessive amounts.

MRI scans of the forearm revealed a recurrent tumor measuring 5.4 × 3.9 × 1.9 cm (Additional file 1: Fig. S1). Positron emission tomography-computed tomography (PET-CT) scans excluded systemic metastatic disease. The locally recurrent tumor was completely resected (R0), and the patient received local radiotherapy with 50 Gy.

Another 8 years later, the patient presented with the left-sided hemiparesis that led to the most recent admission via the emergency department. The patient presented with a Karnofsky Performance Scale (KPS) score of 40. He had average weight and reduced general condition. A cardiac exam showed a regular rate and no murmur. A pulmonary exam showed a vesicular breath murmur. His temperature was 37.0 °C, blood pressure was 116/46 mmHg, and pulse was 99 beats/minute The respiratory rate was 13/minute, peripheral oxygen saturation was 95%, and the Glasgow Coma Scale was 15. The patient was alert, attentive, and oriented. The speech was clear and fluent, with good repetition, comprehension, and naming. The cranial nerve exam did not reveal any abnormalities. Examination of motor skills showed paresis of the left arm proximal 2/5, the left arm distal 4/5, and the left leg 3/5. The gait was not testable due to paresis. The stance was only with maximum support. The laboratory findings revealed elevated leukocytes (12.3 ×10^9^/l) and elevated thrombocytes (437 ×10^9^/l). The daily medication was folic acid 5 mg (1-0-0), calcium carbonate/cholecalciferol (1-0-0), and Novamin (oral drops) (40-0-40).

**Fig. 1:**
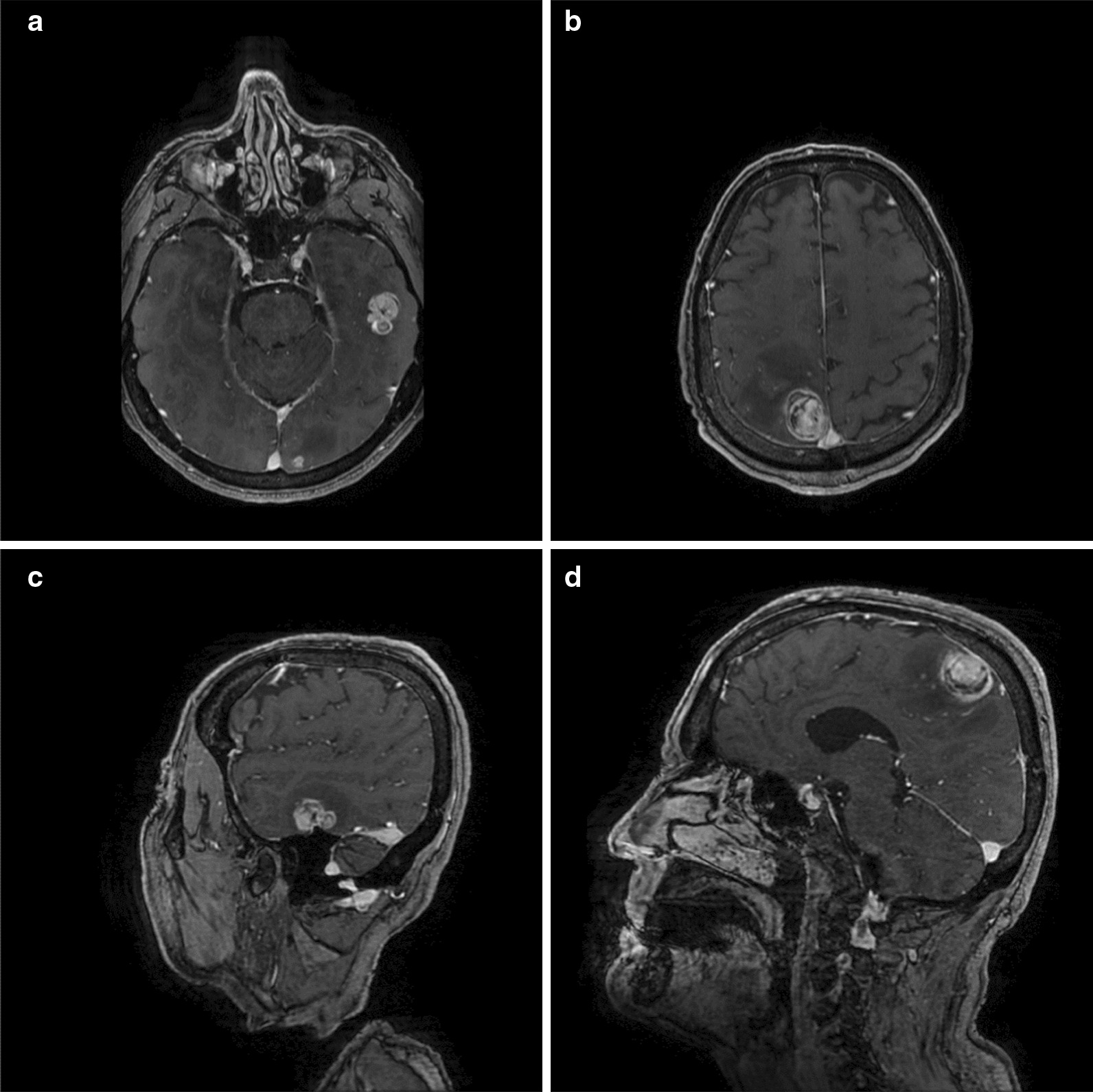
Contrast-enhanced T1-weighted magnetic resonance imaging scans demonstrating left temporal (**a**, **c**) and right parietal (**b**, **d**) metastases of leiomyosarcoma (**a**+**b** axial; **c**+**d**: sagittal)

Preoperative MRI scans of the head confirmed multiple contrast-enhancing mass lesions (Fig. 1). Microsurgical resection of the right parietal mass was performed. The left temporal mass lesion was left unaddressed since a complete resection of all masses was impossible without an increased risk of aphasia. CT scans revealed further metastasis-suspect foci in the pancreas and the first sacral vertebral body.

One month after neurosurgical resection, the patient suffered from melena, and a gastroduodenoscopy was performed. An antral swelling with oozing bleeding was found, and biopsy revealed a third metastasis of the known leiomyosarcoma. After good recovery, the patient received whole-brain irradiation with 30 Gy and local irradiation of the metastasis in the first sacral vertebra with 30 Gy. The patient showed functional recovery with discharge home possible. He refused adjuvant chemotherapy. The patient died 3 months after the neurosurgical intervention and 9 years after the first diagnosis of leiomyosarcoma due to systemic progression. An autopsy was not performed.

### Histopathological data

The first resection specimen derived from the right forearm displayed moderate cell density, with spindle-shaped cells arranged in intersecting fascicles located in the deep subcutaneous fibrous tissue. The tumor cells displayed a focally increased degree of pleomorphism, few visible mitotic figures, and small areas of fibrinoid necrosis. Immunohistochemical analysis revealed that the cells were positive for sarcomere actin, and Ki67 labeled around 10% of the tumor cells as proliferative. The tissue was classified as leiomyosarcoma grade 1, according to the FNCLCC [Fédération Nationale des Centres de Lutte Contre Le Cancer] grading system.

On recurrence 1 year later, increased cellularity, highly elevated mitotic activity (> 20 per 10 high-power fields [HPF]), and an additional population of pleomorphic, partially multinucleated osteoclast-like giant tumor cells were noted. The recurrent tumor was classified as leiomyosarcoma grade 3 (score 6), according to FNCLCC, rpT1b Nx M0, L0 V0 Pn0, according to the TNM classification.

**Fig. 2:**
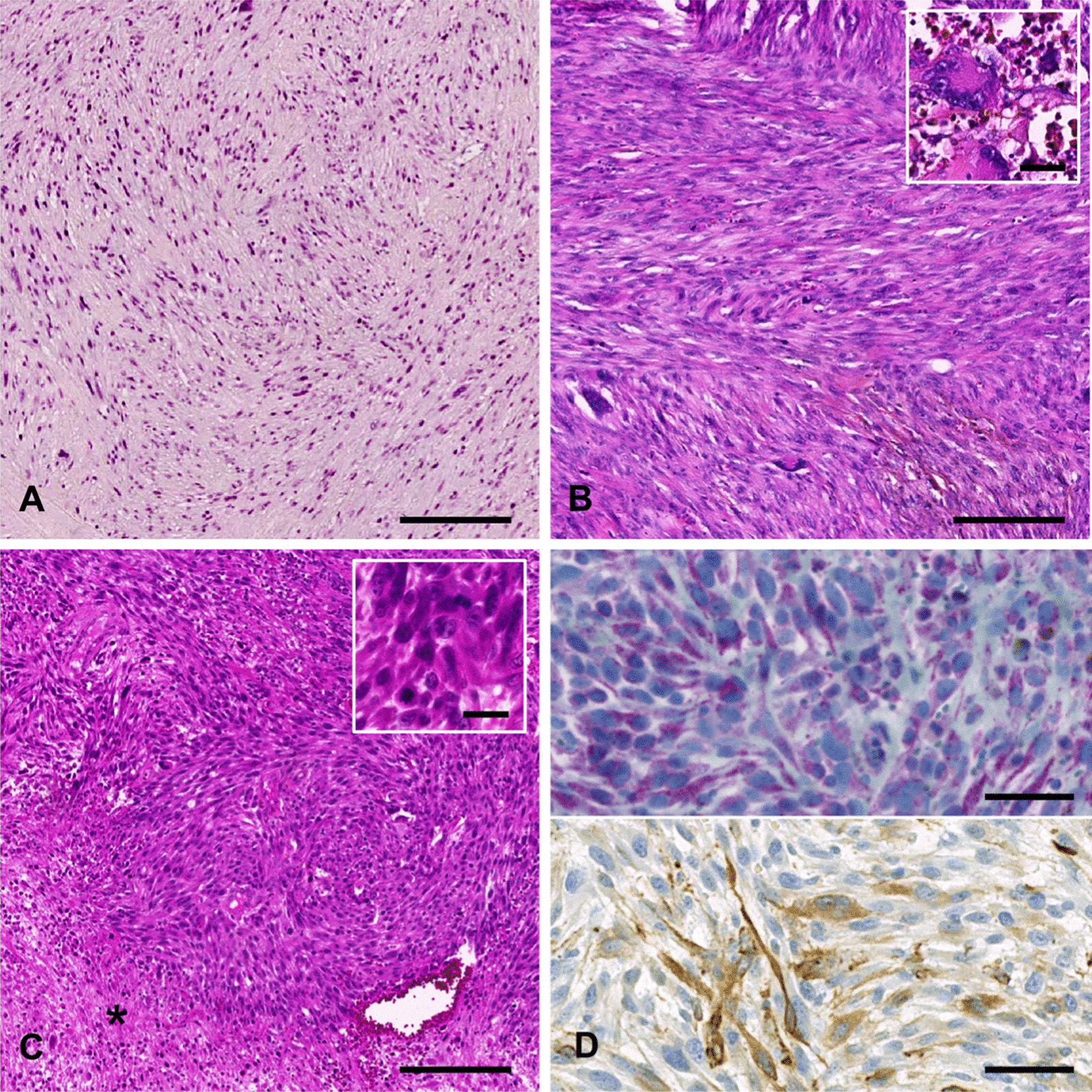
**a** Initial tumor (hematoxylin and eosin stain [HE]): moderately increased density of spindle-shaped cells in a fascicular arrangement. **b** Local recurrence 1 year later (HE): high cellularity, increased mitotic activity, and a high degree of pleomorphism, with the occurrence of bizarre giant cells (inset). **c** Brain metastasis 9 years later (HE): high cell density, fascicular tissue of slightly pleomorphic, spindle-shaped tumor cells, elevated mitotic activity (inset) and intratumoral necrosis (*) **d** Immunohistochemical staining of the brain metastasis: positivity for alpha smooth muscle actin (**d**, upper panel), and sarcomere actin (**d**, lower panel). Scale bars on **a**, **b**, **c**: 200 µm, on insets: 20 µm, on **d**: 50 µm

Resection specimen of the brain metastasis revealed moderate cell density of spindle-shaped, only slightly pleomorphic tumor cells arranged in intersecting fascicles. Giant osteoclast-like cells were absent in the brain metastasis. Mitotic count was highly elevated (>40 per 10 HPF), and necrotic areas constituted around 30% of the entire tissue. On immunohistochemical analysis, the tumor cells displayed patchy reactivity for both alpha smooth muscle actin (α-SMA) and sarcomere actin. In summary, the tumor was diagnosed as a metastasis from the formerly known leiomyosarcoma, FNCLCC grade 3 (score 6) (Fig. 2).

## Discussion

To the best of our knowledge, this is the first case of multiple intracerebral metastases of a non-uterine leiomyosarcoma. Only one other case of a non-uterine leiomyosarcoma has been published, in which a single right frontal subcortical cerebral metastasis was detected in a patient with a right triceps muscle leiomyosarcoma.

Sarcomas of adult patients are a rare entity of malignant tumors, accounting for only 1% of all adult malignancies. In 2017, about 12,390 new cases and 4990 deaths due to sarcomas were described in the United States [2]. Since the sub-entity of a non-uterine leiomyosarcoma is even less frequent, and intracerebral metastasis of these tumors has only been described twice, including this report, the clinical management and adjuvant therapy remain based on individualized decision-making [3].

The mean overall survival after diagnosis of intracranial metastasis of leiomyosarcoma is reported to be 3–18 months. The preoperative Karnofsky Performance Score (KPS) plays a pivotal role in the prediction of median survival (KPS > 60: 12.8 months; KPS < 60: 5.4 months) [3].

For the treatment of advanced leiomyosarcoma, the chemotherapy regimen comprises doxorubicin, ifosfamide, and dacarbazine [4]. The evaluation of several other regimens, such as eribulin, pazopanib, trabectedin, and taxanes, did not result in an enhanced survival rate. Up to now, no clear molecular target has been identified for targeted therapies.

Genomic and transcriptomic analysis of leiomyosarcoma has revealed frequent inactivation of the well-described tumor suppressors TP53 and RB1. Furthermore, substantial mutational heterogeneity, frequent whole-genome duplications, and genomic instability have been detected. In one cohort, up to 57% of the tumors exhibited alterations of phosphatase and tensin homolog (PTEN), a down-regulator of the PI3K-Akt-mTOR signaling axis. Thus, targeting Akt via small-molecule inhibitors has been proposed as a potential novel therapeutic strategy [1].

A further potential target has been revealed by genomic "scarring," which indicates an impaired homologous recombination repair of DNA double-strand breaks. Poly [ADP-ribose] polymerase 1 (PARP-1) inhibitors, which are already used in breast, ovarian, and prostate cancer, could establish a novel therapeutic pathway. Nevertheless, as for the aforementioned Akt signaling inhibition, further mechanistic evaluations of these signaling pathways are awaited.

The management of intracranial sarcoma metastasis includes surgical resection, radiotherapy, stereotactic radiosurgery, and chemotherapy. Since complete resection of all intracranial metastases was not possible in this case, the patient underwent whole-brain irradiation after neurosurgery, but unfortunately did not agree to the proposed chemotherapy (doxorubicin and ifosfamide).

Doxorubicin, which was introduced already in the 1970s, still remains the most effective chemotherapy for advanced sarcomas. Novel combination therapies have recently been explored in clinical trials. The combination of olaratumab, a platelet-derived growth factor receptor alpha (PDGFRα) antibody, with doxorubicin showed no additional benefit in the phase III ANNOUNCE trial [5].

Pazopanib and regorafenib, which represent multikinase small-molecule inhibitors, are further promising agents. Here, the phase II REGOSARC trial showed enhanced progression-free survival in patients with advanced non-adipocytic sarcomas under regorafenib treatment, along with an acceptable safety profile [6].

The prognosis of leiomyosarcoma depends on the histological grade, tumor size, and tumor depth. These factors apply to all leiomyosarcoma, regardless of uterine or non-uterine localization [7]. Tumor size and bone or neurovascular involvement, combined with higher grading, are associated with poor outcomes. Interestingly, leiomyosarcoma of the extremities exhibits a better outcome when compared to retroperitoneal leiomyosarcoma [8].

## Conclusion

Cerebral metastasis of a non-uterine leiomyosarcoma is an extremely rare disease. The treatment has to be based on a multidisciplinary, individualized decision, but the mainstay remains neurosurgical resection, adjuvant radiotherapy, and subsequent administration of doxorubicin.

## Supplementary Information

**Additional file 1: Figure S1.** Positron emission tomography-computed tomography imaging showing an increase in FDG uptake in the tumor (arrow) of the right forearm (A axial, B coronal)

## Data Availability

All the data supporting our findings are contained within the manuscript.

## Abbreviations

Akt: Protein kinase B
FNCLCC: Fédération Nationale des Centres de Lutte Contre Le Cancer
HE: Hematoxylin and eosin staining
HPF: High-power field
KPS: Karnofsky performance score
MRI: Magnetic resonance imaging
mTOR: Mammalian target of rapamycin
PARP-1: Poly [ADP-ribose] polymerase 1
PDGFRα: Platelet-derived growth factor receptor alpha
PET-CT: Positron emission tomography-computed tomography
PI3K: Phosphoinositide 3-kinase
PTEN: Phosphatase and tensin homolog
RB1: Retinoblastoma protein
SMA: Smooth muscle actin
TP53: Tumor protein p53
