# Supplementary material for: A late systemic and brain metastasis from subcutaneous leiomyosarcoma of the right forearm: a case report and review of the literature
Source: J Med Case Rep. 2021 Jan 19;15:14. doi: 10.1186/s13256-020-02625-0 (PMC7814649; doi:10.1186/s13256-020-02625-0)
Supplement: Supplementary file 1 — Additional file 1: Figure S1. Positron emission tomography-computed tomography imaging showing an increase in FDG uptake in the tumor (arrow) of the right forearm (A axial, B coronal) [file 13256_2020_2625_MOESM1_ESM.docx]

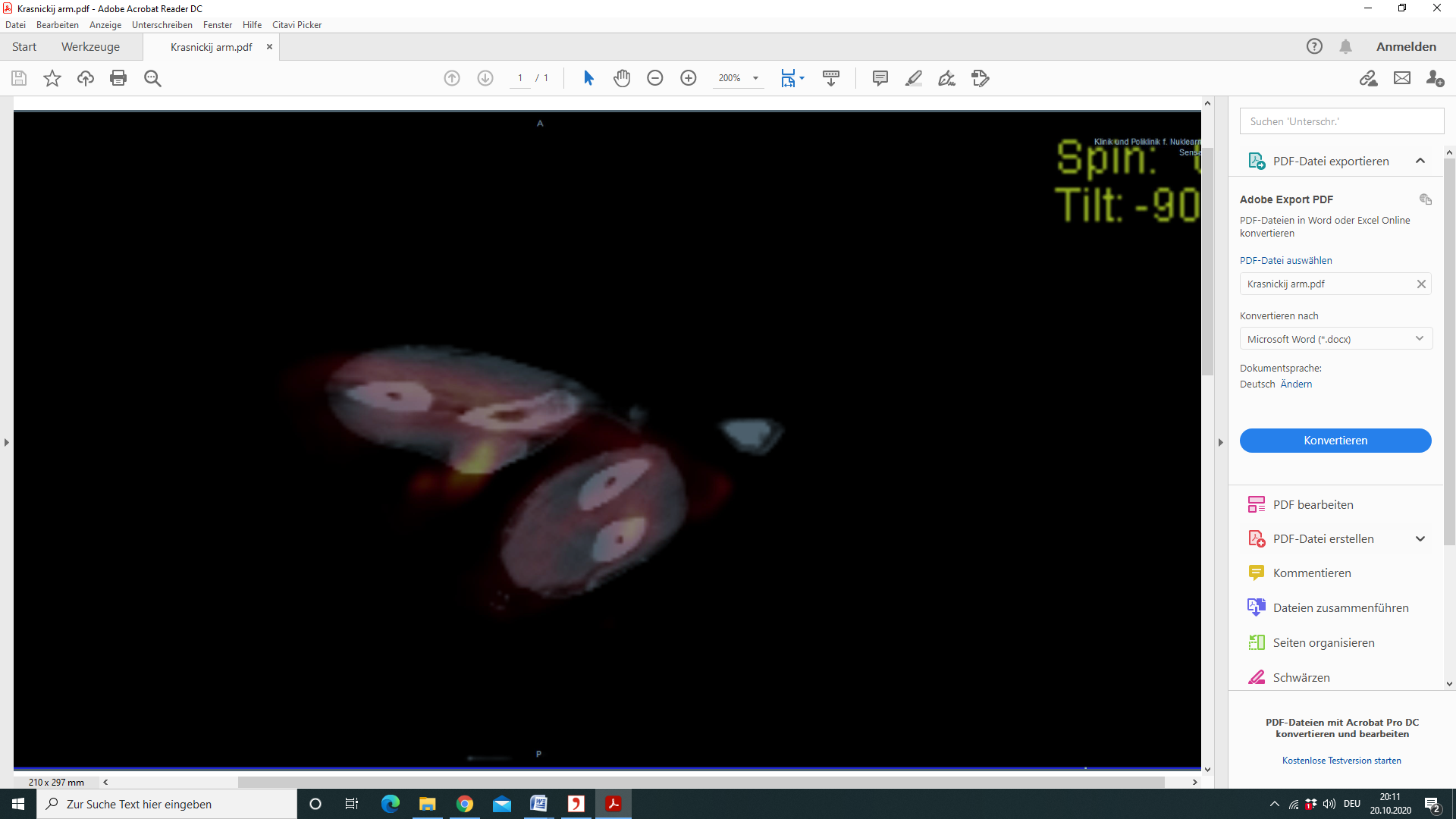

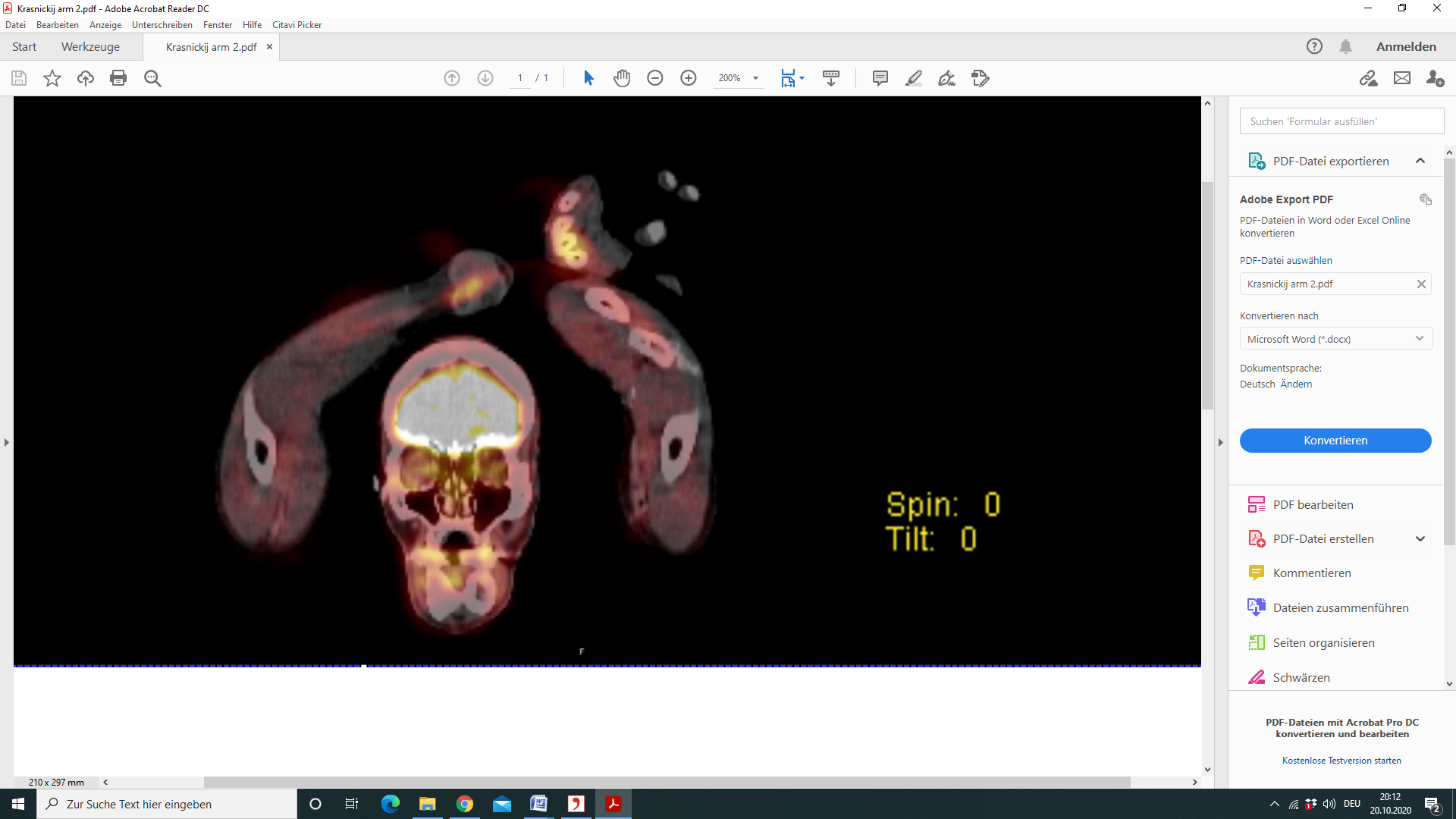


B

A

Additional Figure 1. PET-CT showing an increase FDG uptake in the tumor (arrow) of the right forearm (A axial, B coronar).
